# The correlation between femoroacetabular impingement and superior retinacular artery interruption

**DOI:** 10.1097/MD.0000000000012400

**Published:** 2018-09-21

**Authors:** Liangliang Cheng, Dewei Zhao, Benjie Wang, Xing Qiu, Zihua Wang

**Affiliations:** a Southern Medical University, Guangzhou; b Department of Orthopaedics, Affiliated Zhongshan Hospital of Dalian University, Dalian, Liaoning Province, China.

**Keywords:** femoral head, femoroacetabular impingement, ischemia, osteonecrosis, superior retinacular arteries

## Abstract

The aim of this study was to investigate the relationship between femoroacetabular impingement (FAI) and superior retinacular artery interruption using plain radiographs and digital subtraction angiography (DSA).

Sixty-one patients included in this study were divided into 2 groups based on the superior retinacular artery integrity as evaluated on DSA images. Group A included intact arteries: 33 patients (24 men, 9 women,); group B included interrupted arteries: 28 patients (21 men, 7 women). The parameters of abnormal radiographic findings thought to be associated with FAI, including positive crossover or figure-of-eight sign of acetabulum, lateral center edge angle (LCEA) >40°, Tönnis angle <0°, positive posterior wall sign, alpha angle >50°, and coxa profunda, were evaluated in all patients through plain radiographs.

The cross-over sign (Group A: 0, Group B: 8, *P* = .0035), LCEA (Group A: 1, Group B: 7, *P* = .0190), Tönnis angle (Group A: 3, Group B: 13, *P* = .0026), and alpha angle (Group A: 7, Group B: 17, *P* = .0039) differed significantly between both groups. However, there were no statistically significant differences in posterior wall sign (Group A: 9, Group B: 12, *P* = .3143) or coxa profunda (Group A: 12, Group B: 8, *P* = .7096).

Patients with interrupted blood supply of the superior retinacular arteries displayed more parameters of abnormal radiographic findings associated with FAI thereby indicating potential correlation between FAI and the interruption of superior retinacular arteries.

## Abstract 2

Supplemental Digital Content is available in the text

## 1 Introduction

Osteonecrosis of the femoral head (ONFH) is a common refractory disease in the field of orthopedics. The etiology of osteonecrosis is still unclear. Current theories include lipid metabolism disorder theory, intravascular coagulation theory, bone cell apoptosis theory, gene regulation theory, etc. Although there is controversy among various theories. It is generally believed by majority of scholars that microcirculatory disorders in the femoral head are the ultimate pathway for osteonecrosis. ONFH may result from a host of etiologies, the most common of which is arterial ischemia caused by intravascular coagulation and thrombocyte aggregation.^[1–3]^ It is the interruption or damage of blood supply to the femoral head, causing death and subsequent repair of bone cells and bone marrow components, which in turn leads to structural changes in the femoral head, collapse of the femoral head, and joint dysfunction. Therefore, it is important to understand the blood supply to the femur and the factors that may cause damage to the blood supply.

In adults, the main blood supply of the femoral head is the deep branch of the medial femoral circumflex artery.^[4,5]^ The medial femoral circumflex artery perforates the joint capsule from the cranial border of the musculus gemellus superior tendon and then runs intracapsularly along the dorsolateral aspect of the femoral neck to reach the cartilage-bone junction at the anterolateral aspect of the femoral neck, where it perforates the cortical bone with 2 to 7 terminal branches, also termed “superior retinacular arteries.”^[4,5]^ Lavigne et al^[6]^ showed that 80% of all vascular foramina of the retinacular vessels at the head-neck junction are located in the anterosuperior and posterosuperior regions of the femoral head-neck junction. Coincidentally, the position of impact of femoroacetabular impingement (FAI) is located in the same regions of the femoral neck.^[7]^ Therefore, is FAI related to interruption of the superior retinacular arteries? In this study, digital subtraction angiography (DSA) was used to study the relationship between FAI and superior retinacular artery interruption.

## 2 Methods

Between January 2013 and December 2017, 223 consecutive patients underwent hip-preserving operations (including core decompression, vascularized bone flap implantation, tantalum rob implantation) in our department because of ONFH. The patients were diagnosed according to X-rays, computed tomography (CT) or magnetic resonance imaging (MRI) examination, and pathology of the bone scraped away during operation.^[8]^ In addition, during the admission, patients’ height and weight were recorded to calculate the BMI index, and blood tests, including coagulation, platelets, cholesterol, blood lipids, protein C and protein S, would be completed. They also underwent DSA examination. Exclusion criteria were lack of X-ray imaging or X-ray examination was not standard, a history of Legg–Calve–Perthes disease, any risk factor that could have contributed to arterial ischemia such as trauma, chronic corticosteroid use, excessive alcohol consumption (more than 400 mL a day), nicotine consumption (more than 10 cigarettes a day), or any other coagulation disorder (abnormal of blood tests mentioned above). On the basis of the exclusion criteria, of the 223 patients, 61 (45 men and 16 women) were selected, with age 20 to 55 years at the time of surgery. This study was successfully applied for approval to the Ethics Committee of Affiliated Zhongshan Hospital of Dalian University.

The DSA images were evaluated by 2 doctors (LLC, BJW). All the images were independently evaluated by 2 doctors. Patients were divided into 2 groups by the integrity of the superior retinacular artery: intact retinacular arteries (group A) and interrupted retinacular arteries (group B).

### 2.1 Radiographic examination

All patients had a standardized anteroposterior pelvis radiographs taken before DSA examination, which were obtained with the patient lying supine on the X-ray table with both lower extremities oriented 15° internally in order to maximize the length of the femoral neck.^[9]^ The radiographer, who had undergone specific training for this examination, ensured the patients remained in the correct posture during the exposures. A film-focus distance of 1.2 m with the beam was used. On an adequately positioned anteroposterior pelvis with appropriate pelvic tilt, the distance between the superior border of the pubic symphysis and the sacrococcygeal joint should measure 3 to 5 cm.^[7,10]^ All images were reviewed by 2 blinded observers (LLC, BJW) independently. Some parameters of abnormal radiographic findings deemed to be associated with FAI were measured with previously described methods^[9,11]^ and they included a positive crossover or figure-of-eight sign of the acetabulum, lateral center edge angle (LCEA) >40°, Tönnis angle <0°, a positive posterior wall sign, alpha angle >50°, and coxa profunda.

### 2.2 Statistical analysis

Analysis was performed using SPSS software (version 23; IBM Corp., Armonk, NY). Descriptive statistics using cases numbers and mean value ± standard deviation. Comparison between the 2 groups was conducted using *t* test for continuous variables and the Chi-square test for categorical variables.

Two blinded reviewers (LLC and BJW) independently reviewed the DSA images and X-ray images of the 61 patients on 2 separate occasions; there was no communication between the reviewers. The images were presented to reviewers in a random order, and presentation orders were changed for repeat sessions. Interobserver and intraobserver reliabilities of the prevalences of abnormal radiographic findings associated with FAI and the integrity of superior retinacular artery were assessed using correlation coefficients. Intraclass correlation coefficients were interpreted as follow: <0.20, slight agreement; 0.21 to 0.40, fair agreement; 0.41 to 0.60, moderate agreement; 0.61 to 0.80, substantial agreement; and >0.80, almost perfect agreement.^[12]^ All reported *P* values are 2-sided, and *P* value <.05 was considered significant.

## 3 Results

The patients’ demographic data showed that there was no statistical difference in age, gender, body mass index (BMI), and Association Research Circulation Osseous (ARCO) stage in the 2 groups (Table 1). The detailed data was in the Supplementary material.

**Table 1:**
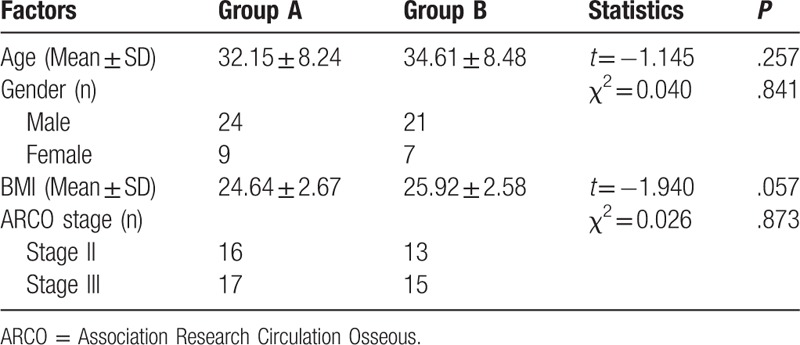
Comparison of patients’ demographic data.

Intraobserver and interobserver correlations for combinations of all measurements were found to be reproducible and reliable among observers (Table 2).

**Table 2:**
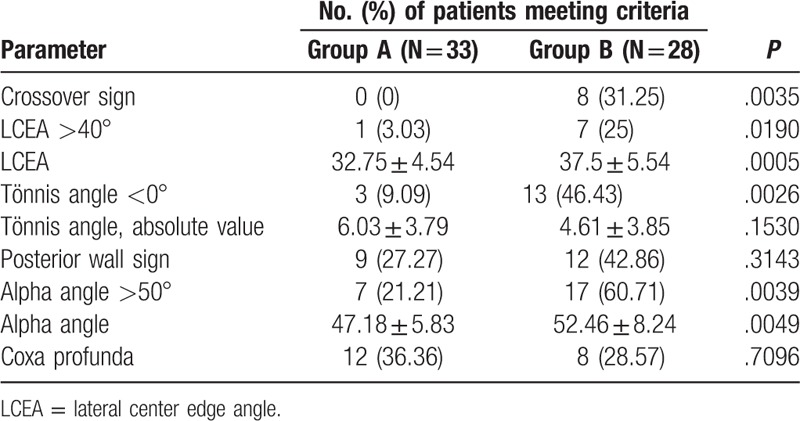
The number of patients with different parameters.

**Figure 1:**
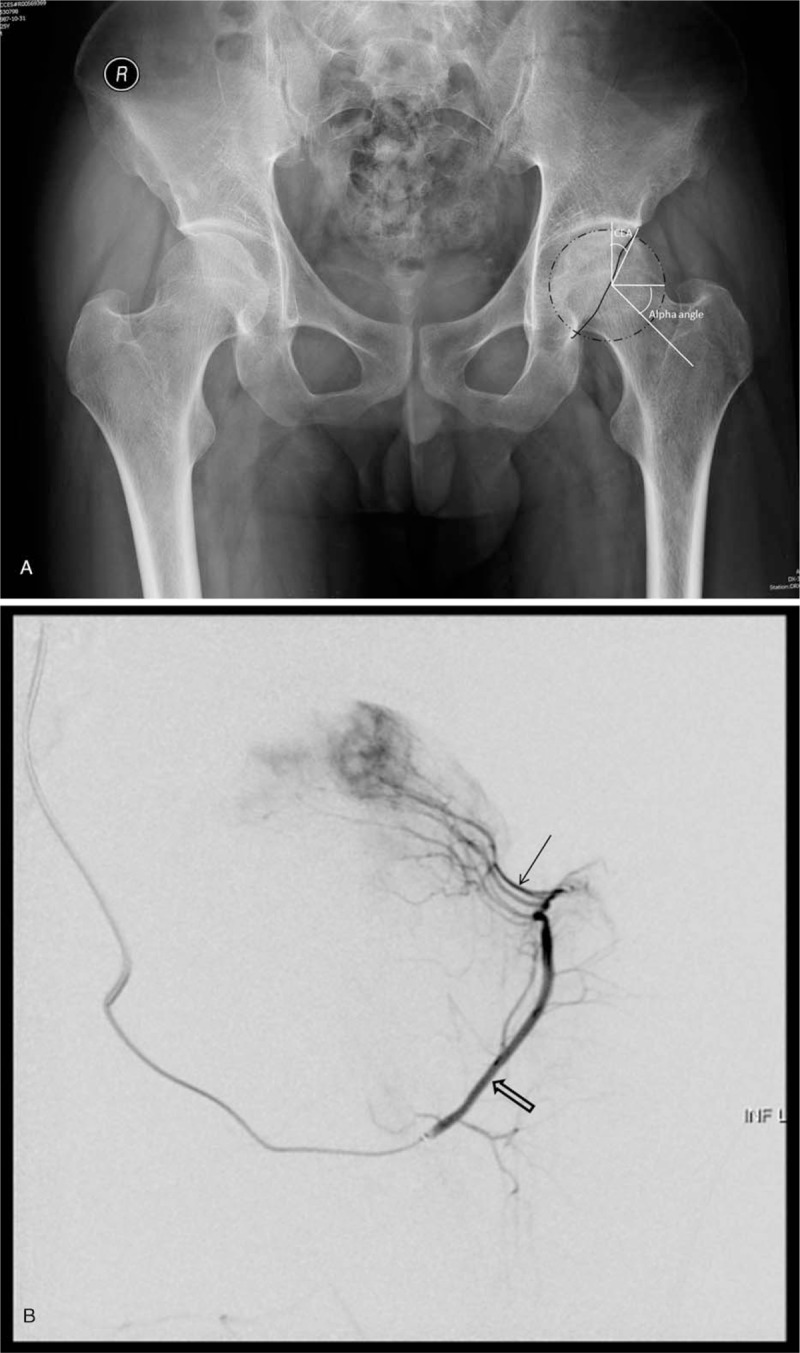
(A) X-ray image of a 35-year-old male patient. (B) DSA examination of the same patient in (A).

**Figure 2:**
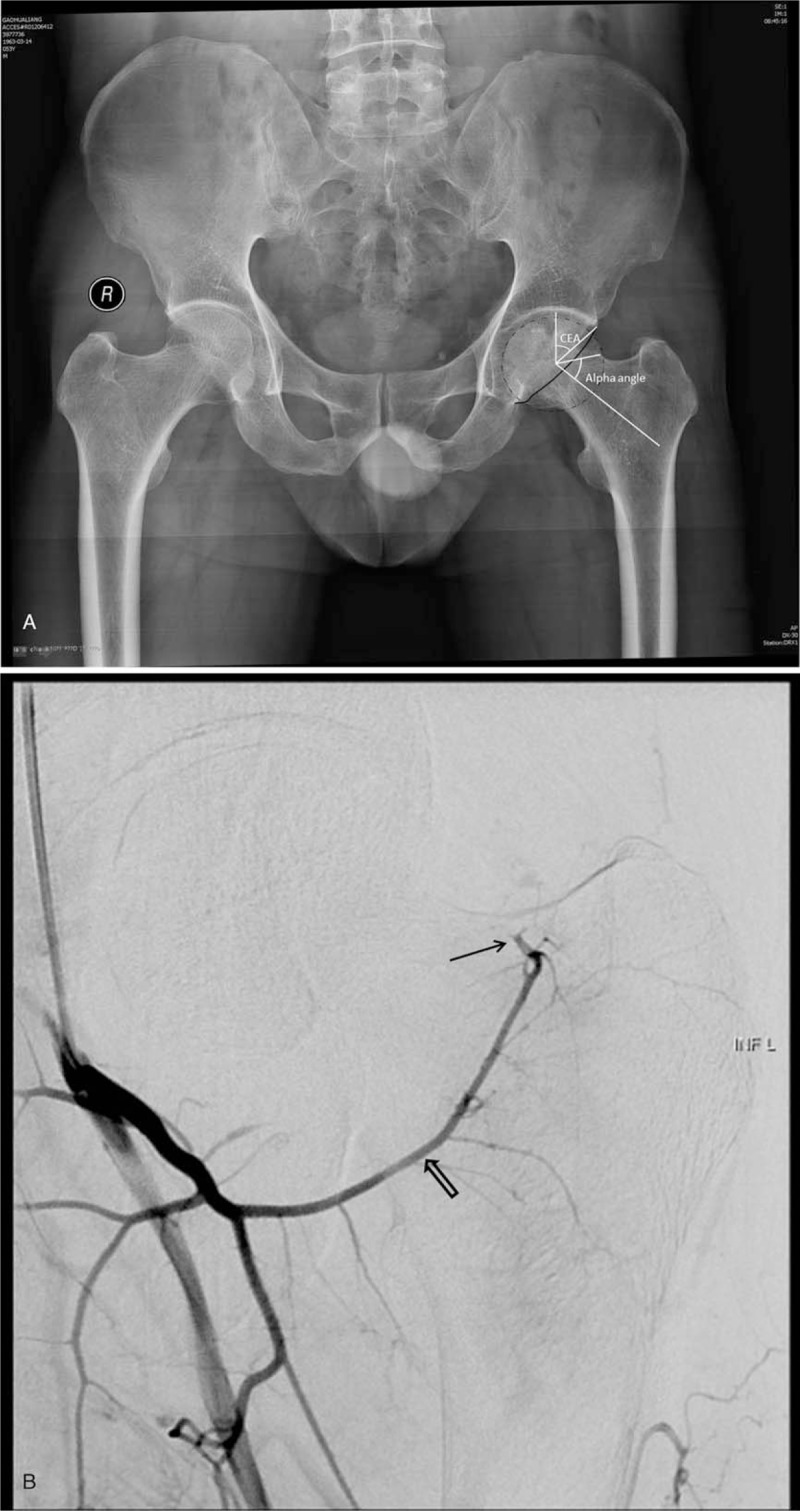
(A) X-ray image of a 20-year-old male patient. (B) DSA examination of the same patient in (A).

The crossover sign (*P* = .0035), LCEA (*P* = .0190), Tönnis angle (*P* = .0026), and alpha angle (*P* = .0039) differed significantly between the 2 groups. The patients in Group A, whose superior retinacular arteries were intact, showed less of these parameters (Figure 1). The patients in group B, whose superior retinacular arteries were interrupted, showed more of these parameters (Figure 2). However, there were no statistically significant differences in the posterior wall sign (*P* = .3143) or coxa profunda (*P* = .7096). The number of patients with each parameter and the results of the statistical analyses are summarized in Table 3.

**Table 3:**
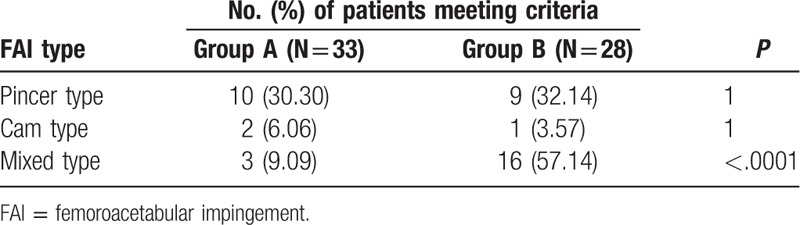
The number of patients in different FAI types.

In the case of FAI category, there were 10 cases of pincer-type, 2 cases of cam-type, and 3 cases of mixed-type in group A, while there were 9 cases of pincer-type, 1 case of cam-type, and 16 cases of mixed-type in group B. There was a statistically significant difference between the 2 groups of mixed-type (Table 4).

**Table 4:**
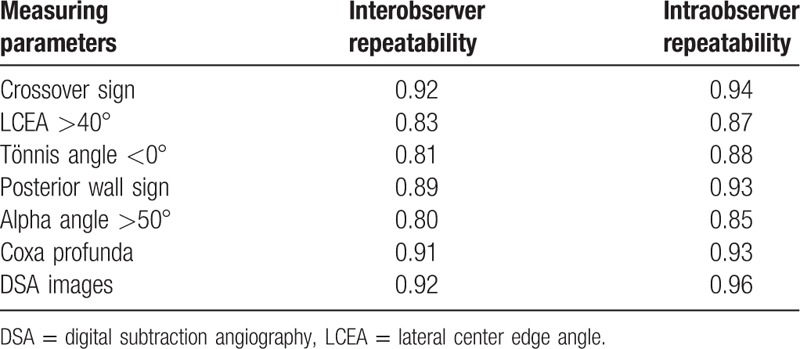
Interobserver and intraobserver reliabilities of radiographic parameters of X-rays and DSA images.

## 4 Discussion

In this study, patients with interrupted superior retinacular artery blood supply displayed more abnormal radiographic findings related to FAI, such as positive crossover sign, >40 degrees of LCEA, <0° of Tönnis angle, and >50° of alpha angle. The results showed that the continuity of superior retinacular artery blood supply might be associated with anatomical abnormalities of FAI. Our study suggested that superior retinacular artery blood supply might be associated with the local pressure increase caused by anatomical abnormalities, which was commonly found in mixed-type FAI. It was speculated that femur and acetabulum of mixed-type FAI worked together on superior retinacular artery, causing the surrounding tissue to produce a higher pressure, which caused endothelial damage through repetitive press and formed thrombus to clog up blood vessels.

Fraitzl et al^[13]^ found a higher frequency of cam-type head-neck junctions in patients with osteonecrosis and proposed that superior retinacular arteries may be pinched by the impingement of the femoral neck and acetabulum. But only 1 indicator, alpha angle, was evaluated in this study, and imaging indicators related to pincer-type were not evaluated. Therefore, there might be many mixed-type FAIs among his patients, which was similar to our findings. Our angiography results could more directly reveal the condition of the blood supply, which further supported his hypothesis. Nötzli et al^[14]^ studied the blood supply of the femoral head in different positions and found important variations; results indicated that the blood flow of the medial femoral circumflex artery might be affected by impingement of the acetabulum and femoral neck and the greatest reduction of blood flow was observed in external rotation, when the posterosuperior head-neck junction was compressed against the acetabular rim. This supports our results, as we found that interruption of the blood supply of the superior retinacular artery more easily occurred in patients with abnormal radiographic findings related to FAI, especially in mixed-type FAI.

Ogden^[15]^ found that the blood supply to the femoral head of infants and young children gradually changed after birth. At the time of birth, the first part of the femoral head of the baby was fed by the branch of lateral circumflex femoral artery. With the growth and development of the baby, the branch of the lateral circumflex femoral artery in the first half of the femoral head gradually degenerates or even disappear, and is replaced by the terminal branch of the medial femoral artery (upper support artery).^[15]^ Ogden^[15]^ believes that the degeneration or disappearance of the lateral femoral artery branch is due to the pressure on the blood vessels caused by the tendon of the iliopsoas and the iliofemoral ligament in front of the hip joint.^[15]^ This is consistent with the hypothesis of our study. But will the blood vessels suffer from long-term repeated external pressures that will degenerate or disappear? This issue needs further research to confirm.

There are also limitations to the current study. First, all patients had ONFH. Although exclusion criteria could exclude cases with thrombembolia caused by abnormal blood coagulation, we may have been unaware of other potential causes of blood supply interruption. Second, if X-ray projection angles show deviation, errors would occur in measuring abnormal radiographic findings related to FAI. Third, all the patients suffered femoral head necrosis, and there might be selection bias in the selection of patients, without a control group of normal hip joint patients. The problem will be further improved in the future. Fourth, this was a retrospective study conducted at a single center, which limits the extrapolation of our results. Multicenter, prospective studies are needed to further confirm the results of this study.

## 5 Conclusion

In this study, potential correlations between FAI and the interruption of superior retinacular arteries were indicated that did necessarily imply causality. Accordingly, larger multicenter cohorts and anatomical study should be done to confirm these findings.

## Acknowledgment

We thank our colleagues of Affiliated Zhongshan Hospital of Dalian University, for their critical comments and kind help.

## Author contributions

**Conceptualization:** Dewei Zhao, Liangliang Cheng.

**Data curation:** Liangliang Cheng, Benjie Wang.

**Formal analysis:** Liangliang Cheng, Xing Qiu.

**Funding acquisition:** Dewei Zhao.

**Investigation:** Xing Qiu.

**Validation:** Zihua Wang.

**Writing - original draft:** Liangliang Cheng, Zihua Wang.

**Writing - review & editing:** Dewei Zhao.

## Supplementary Material

Supplemental Digital Content
