# The correlation between femoroacetabular impingement and superior retinacular artery interruption: Erratum

**DOI:** 10.1097/MD.0000000000012963

**Published:** 2018-10-19

In the article, “The correlation between femoroacetabular impingement and superior retinacular artery interruption”,^[1]^ which appears in Volume 97, Issue 38 of *Medicine*, Tables 2, 3 and 4 were numbered incorrectly. The correctly numbered tables are below.

**Table 2:**
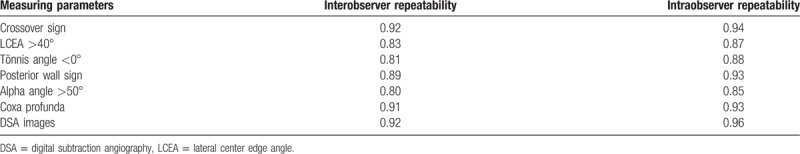
Interobserver and intraobserver reliabilities of radiographic parameters of X-rays and DSA images.

**Table 3:**
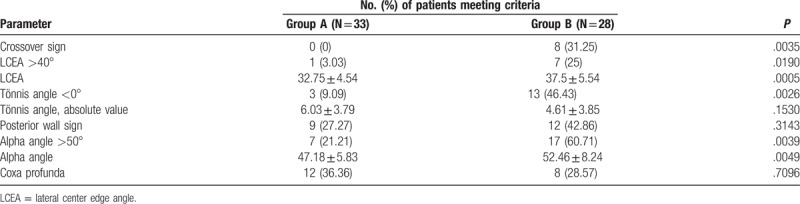
The number of patients with different parameters.

**Table 4:**
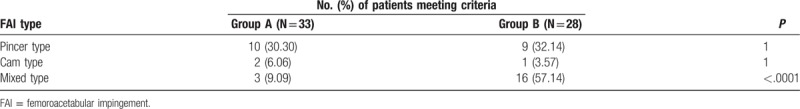
The number of patients in different FAI types.
