# Supplementary material for: The correlation between femoroacetabular impingement and superior retinacular artery interruption
Source: Medicine (Baltimore). 2018 Sep 21;97(38):e12400. doi: 10.1097/MD.0000000000012400 (PMC6160198; doi:10.1097/MD.0000000000012400)
Supplement: Supplemental Digital Content [file medi-97-e12400-s001.docx]

**supplementary Table 1: Body mass index**

| Group | No. | Age | Gender | BMI | ARCO |
| --- | --- | --- | --- | --- | --- |
| A | 1 | 33.00 | F | 22.30 | 2 |
| A | 2 | 37.00 | M | 23.53 | 3 |
| A | 3 | 22.00 | M | 30.36 | 3 |
| A | 4 | 29.00 | M | 21.63 | 3 |
| A | 5 | 26.00 | M | 23.71 | 2 |
| A | 6 | 36.00 | F | 19.74 | 2 |
| A | 7 | 40.00 | F | 31.50 | 3 |
| A | 8 | 38.00 | M | 23.73 | 3 |
| A | 9 | 31.00 | F | 25.09 | 3 |
| A | 10 | 48.00 | M | 24.82 | 2 |
| A | 11 | 30.00 | M | 27.95 | 2 |
| A | 12 | 27.00 | F | 24.48 | 3 |
| A | 13 | 25.00 | M | 23.58 | 3 |
| A | 14 | 38.00 | M | 26.22 | 3 |
| A | 15 | 22.00 | M | 27.77 | 2 |
| A | 16 | 21.00 | M | 22.42 | 2 |
| A | 17 | 32.00 | M | 27.23 | 2 |
| A | 18 | 25.00 | M | 26.07 | 3 |
| A | 19 | 38.00 | F | 23.66 | 2 |
| A | 20 | 22.00 | M | 26.26 | 2 |
| A | 21 | 42.00 | M | 23.30 | 3 |
| A | 22 | 44.00 | M | 18.90 | 3 |
| A | 23 | 28.00 | M | 26.55 | 2 |
| A | 24 | 27.00 | F | 26.32 | 2 |
| A | 25 | 37.00 | M | 25.20 | 3 |
| A | 26 | 23.00 | F | 27.39 | 2 |
| A | 27 | 19.00 | M | 23.68 | 2 |
| A | 28 | 23.00 | M | 21.59 | 3 |
| A | 29 | 48.00 | M | 23.15 | 2 |
| A | 30 | 36.00 | F | 24.34 | 3 |
| A | 31 | 32.00 | M | 24.08 | 3 |
| A | 32 | 37.00 | M | 23.15 | 2 |
| A | 33 | 45.00 | M | 23.46 | 3 |
| B | 1 | 48.00 | M | 27.18 | 3 |
| B | 2 | 38.00 | M | 25.55 | 3 |
| B | 3 | 43.00 | F | 28.56 | 2 |
| B | 4 | 43.00 | M | 23.77 | 3 |
| B | 5 | 32.00 | M | 20.97 | 3 |
| B | 6 | 42.00 | M | 27.28 | 2 |
| B | 7 | 29.00 | F | 25.67 | 3 |
| B | 8 | 34.00 | M | 19.90 | 2 |
| B | 9 | 26.00 | M | 27.89 | 3 |
| B | 10 | 55.00 | M | 28.76 | 2 |
| B | 11 | 27.00 | M | 26.08 | 2 |
| B | 12 | 25.00 | F | 27.39 | 3 |
| B | 13 | 36.00 | M | 28.47 | 3 |
| B | 14 | 28.00 | M | 26.72 | 2 |
| B | 15 | 29.00 | M | 26.31 | 3 |
| B | 16 | 22.00 | M | 21.28 | 2 |
| B | 17 | 44.00 | M | 25.77 | 2 |
| B | 18 | 39.00 | M | 23.45 | 2 |
| B | 19 | 35.00 | M | 24.82 | 3 |
| B | 20 | 20.00 | F | 27.74 | 3 |
| B | 21 | 39.00 | F | 25.54 | 3 |
| B | 22 | 46.00 | F | 26.61 | 2 |
| B | 23 | 35.00 | M | 28.43 | 2 |
| B | 24 | 28.00 | M | 23.60 | 3 |
| B | 25 | 26.00 | M | 31.10 | 3 |
| B | 26 | 30.00 | M | 23.14 | 2 |
| B | 27 | 39.00 | M | 27.55 | 3 |
| B | 28 | 31.00 | F | 27.18 | 2 |
